# Thiourea-Modified TiO_2_ Nanorods with Enhanced Photocatalytic Activity

**DOI:** 10.3390/molecules21020181

**Published:** 2016-02-01

**Authors:** Xiaofeng Wu, Shun Fang, Yang Zheng, Jie Sun, Kangle Lv

**Affiliations:** Key Laboratory of Catalysis and Materials Science of the State Ethnic Affairs Commission & Ministry of Education, South-Central University for Nationalities, Wuhan 430074, China; xiaofengwu0501@163.com (X.W.); fangshun888@gmail.com (S.F.); yangzheng87@hotmail.com (Y.Z.); jetsun@mail.scuec.edu.cn (J.S.)

**Keywords:** photocatalytic degradation, TiO_2_, nanorods, dope, hydroxyl radicals

## Abstract

Semiconductor TiO_2_ photocatalysis has attracted much attention due to its potential application in solving the problems of environmental pollution. In this paper, thiourea (CH_4_N_2_S) modified anatase TiO_2_ nanorods were fabricated by calcination of the mixture of TiO_2_ nanorods and thiourea at 600 °C for 2 h. It was found that only N element was doped into the lattice of TiO_2_ nanorods. With increasing the weight ratio of thiourea to TiO_2_ (*R*) from 0 to 8, the light-harvesting ability of the photocatalyst steady increases. Both the crystallization and photocatalytic activity of TiO_2_ nanorods increase first and then decrease with increase in *R* value, and R2 sample showed the highest crystallization and photocatalytic activity in degradation of Brilliant Red X3B (X3B) and Rhodamine B (RhB) dyes under visible light irradiation (λ > 420 nm). The increased visible-light photocatalytic activity of the prepared N-doped TiO_2_ nanorods is due to the synergistic effects of the enhanced crystallization, improved light-harvesting ability and reduced recombination rate of photo-generated electron-hole pairs. Note that the enhanced visible photocatalytic activity of N-doped nanorods is not based on the scarification of their UV photocatalytic activity.

## 1. Introduction

In recent years, intensive studies have been reported to on the preparation of TiO_2_ due to its potential application in environmental remediation [1,2,3,4,5]. One of the most significant scientific and commercial advances to date has been the development of visible-light active TiO_2_ photocatalytic materials [6]. It is generally recognized that the band gap excitation of TiO_2_ results in the generation of conduction band electrons (e_cb_^−^) and valence band holes (h_vb_^+^), which is followed by generation of various reactive oxidative species (ROSs) such as hydroxyl radicals (•OH) and superoxide radicals (O_2_^−^) [7]. Using O_2_ as an oxidant, a variety of organic compounds can be degraded into CO_2_ and the corresponding inorganic ions due to the attack of the ROSs in solution. However, the efficiency achieved so far with the TiO_2_-based system is still not high enough to enable practical application due to the quick recombination of photo-genreated e_cb_^−^ and h_vb_^+^, without initiating the chemical reactions with surface adsorbates [8].

Recently, great effort has been dedicated to fabrication of 1 dimensional (1D) TiO_2_ nanomaterials such as nanotubes [9,10,11], nanorods [12,13,14,15] nanowires [16] and nanofibers [3,17,18,19,20] due to their unique structures. It is believed that 1D structure can facilitate the quick separation of photo-generated electrons and holes along opposite direction, retarding the recombination of electron-hole pairs, and therefore enhancing the photocatalytic activity of TiO_2_ nanomaterials. They, however, can still only be activated by UV light (band gap 3.2 eV). As a result, visible-light driven 1D TiO_2_ nanomaterials are highly desired.

Doping TiO_2_ with metal or nonmetal atoms is an effective way to extend the absorption of TiO_2_ from UV to the visible region [6,11,21,22,23,24,25,26,27]. For example, Gang *et al.* [27] reported the preparation of the visible light responsive N-doped anatase TiO_2_ sheets with dominant {001} facets by hydrothermal treatment of TiN-HF mixed solution. The study of Manthina *et al.* [28] showed that the band edge positions of the TiO_2_ and ZnO can been shifted by doping with Zr^4+^ and Co^2+^, respectively. Doping moved the conduction band minimum of ZnO to a more positive potential than that of the TiO_2_, enabling electron transfer from dye-sensitized TiO_2_ nanoparticles to the underlying ZnO nanorods for efficient charge collection.

Recently, we have successfully fabricated Bi, C and N co-doped TiO_2_ nanoparticles by a sol-gel method. Although the prepared TiO_2_ nanoparticles are visible-light responsive, they showed poor crystallization [29]. Our group also fabricated C, N and S codoped TiO_2_ hollow microspheres (TiO_2_-HMSs) by calcination of the mixture of TiO_2_-HMSs and cysteine at 300 °C for 2 h [30]. According to the study of Zhang *et al.* [31], the photoreactivity of visible-light responsive C, N and S co-dopd TiO_2_ nanoparticles, prepared by calcination the mixture of TiO_2_ and thiourea at 300 °C, which exhibited stronger photo-absorption in the visible light region and higher dopant content, indicating its potential for higher visible-light photocatalytic activity. However, according to the report of Zhu *et al.* [32], surface hybridization of TiO_2_ particles with graphite-like carbon may be formed during calcination of TiO_2_ in the presence of organic materials, where high migration efficiency of photoinduced electrons at the graphite-like carbon/TiO_2_ interface is response for the enhanced photocatalytic activity.

In this paper, we report the preparation of 1D N-doped TiO_2_ nanorods by simply calcining TiO_2_ nanorods in the presence of thiourea, which is one of the commonly used TiO_2_ dopant precursors [6]. The effect of weight ratio of thiourea to TiO_2_ (*R*) on the structure and photocatalytic activity of TiO_2_ nanorods was systematically studied. To exclude the formation of surface hybridized graphite-like carbon, the calcination temperature was set to 600 °C, because almost all of the organics can be removed by calcination at high temperature under air.

## 2. Results and Discussion

### 2.1. XRD

**Figure 1:**
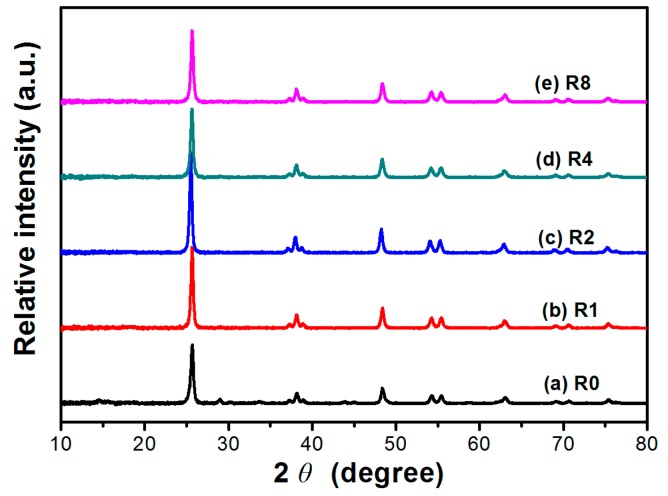
XRD patterns of TiO_2_ nanorods prepared in the presence of different *R* (weight ratio of thiourea to TiO_2_).

XRD was used to investigate the phase structure and crystallite size of the TiO_2_ samples with different dopant levels. Figure 1 shows XRD patterns of the TiO_2_ samples calcined at 600 °C with different mass ratio (*R*) of thiourea to TiO_2_ nanorods. It can be seen that all the samples were pure anatase phase with a peak at 2θ = 25.3° corresponding to the (101) plane diffraction of anatase TiO_2_ [23]. With increasing *R* value from 0 to 2, the peak intensities of anatase increased, indicating an enhancement of crystallization (from 1.0 to 1.69). Meanwhile, the width of the (101) plane diffraction peaks became narrower, showing an increase in anatase crystallite size (from 22.2 to 25.8 nm) (see Table 1). Improved crystallization means less deflects, which favors for the enhanced photocatalytic activity of TiO_2_. However, both the crystallization (from 1.69 to 1.21) and crystalline size (from 25.8 to 22.9 nm) of TiO_2_ samples were found to decrease with further increase in the *R* value from 2 to 8.

**Table 1:** Nitrogen adsorption and XRD characterization results of the photocatalysts.

Sample	*R* ^a^	Pore Volume (cm^3^·g^−1^)	Average Pore Size (nm)	*S*_BET_ (m^2^·g^−1^)	Average Crystalline Size (nm)	Relative Crystallinity ^b^
R0	0	0.095	10.6	35.9	22.2	1
R1	1	0.082	10.5	31.5	25.0	1.37
R2	2	0.114	12.7	35.8	25.8	1.69
R4	4	0.107	13.4	32.0	22.9	1.17
R8	8	0.097	11.4	34.2	22.9	1.21

^a^ Weight ratio of thiourea to TiO_2_ before calcination; ^b^ Relative crystallinity: the relative intensity of the diffraction peak from the anatase (101) plane (R0 is used as reference).

### 2.2. Morphology of Titanates

**Figure 2:**
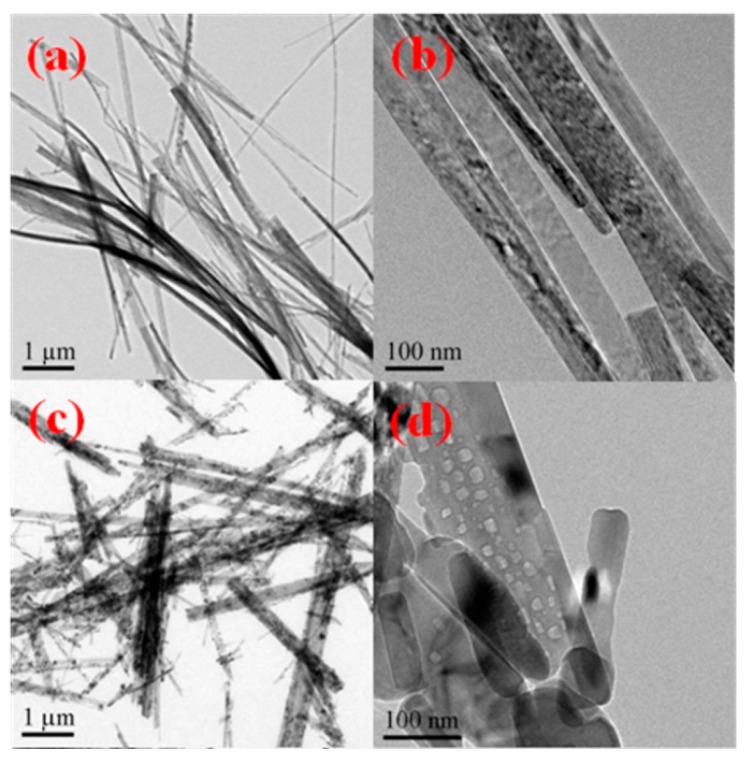
TEM images of sodium titanates before acid wash (**a**,**b**), and anatase TiO_2_ nanorods after hydrothermal treatment by H_2_SO_4_ (**c**,**d**).

Figure 2 shows the TEM images of sodium titanates (Figure 2a,b) and corresponding hydrogen titanates (Figure 2c,d). Consistent with the reports in the literature [16], the prepared sodium titanates are nanowires with length of more than 10 um and diameter of about 50 nm. After treatment with H_2_SO_4_, however, the length of hydrogen titanates decreases to less than 10 um. This is due to acid induced transformation from sodium titanates to hydrogen titanates.

### 2.3. Morphology of Thiourea-Modified TiO_2_ Nanorods

**Figure 3:**
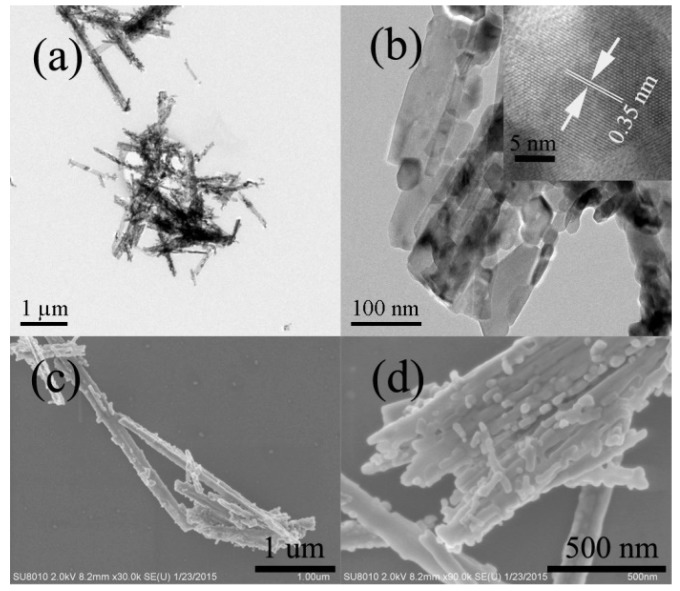
TEM images (**a**,**b**) and SEM images (**c**,**d**) of thiourea-modified TiO_2_ nanorods (R2), Inset of (**b**) showing the high-resolution TEM image of R2 TiO_2_ nanorod.

Figure 3 shows the TEM and SEM images of the thiourea-modified TiO_2_ sample (R2). It can be clearly seen that the morphology of thiourea-modified TiO_2_ sample is similar to hydrogen titanates nanorods precursor. However, carefully view showed that some nanoparticles were also formed after modification (Figure 3b,d), possibly due to the phase transformation from hydrogen titanates to anatase TiO_2_ after calcination at 600 °C. High-resolution TEM image (inset of Figure 3b) clearly shows the lattice spacing of 0.35 nm, corresponding to the (001) planes of anatase TiO_2_, further confirming the high crystallization of R2 TiO_2_ nanorods. Judging from the clearly boundary of TiO_2_ nanocrystals shown in high-resolution TEM image of R2 (inset of Figure 3b), the possibility of the formation of carbon shell (graphitic carbon) can be excluded.

### 2.4. Diffused Reflectance Spectroscopy

**Figure 4:**
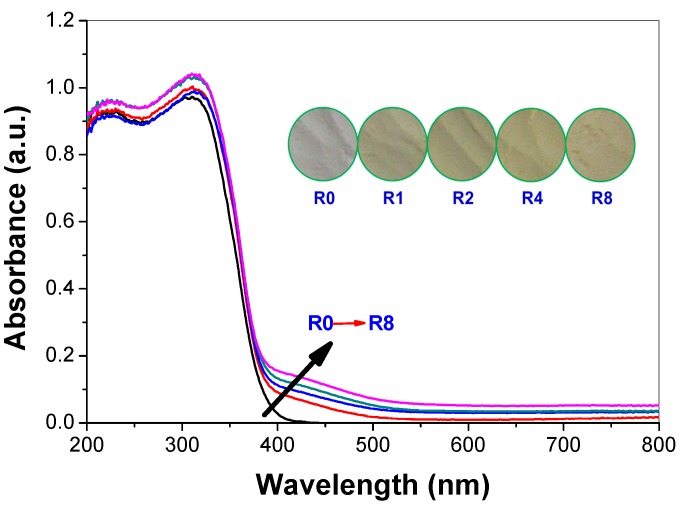
Diffused reflectance spectroscopy and the corresponding optical digital images (inset) of the prepared photocatalysts.

Usually, doping obviously influences light absorption characteristics of TiO_2_ [29]. Therefore, the optical property of the pristine and thiourea-modified TiO_2_ nanorods were measured by UV-vis diffuse reflectance spectra. As displayed in Figure 4, all of these samples display the typical absorption with an intense transition in the UV region of the spectra, which is assigned to the intrinsic band gap absorption of TiO_2_ due to the electron transitions from the valence band to conduction band (O2p → Ti3d) [30]. The pure TiO_2_ (R0) shows no absorption above its fundamental absorption edge (around 400 nm). In contrast, the absorption spectra of the thiourea-modified TiO_2_ samples show enhanced absorption in the visible region. The inset in Figure 4 shows the corresponding photos of pristine and thiourea-modified TiO_2_ samples. It can be seen that the color of TiO_2_ sample becomes deeper with an increase in *R* value, which is consistent with the diffused reflectance spectroscopy. Undoubtedly, these results reveal that the nonmetal elements are indeed incorporated into the lattice of TiO_2_, forming two phase structures (pristine and doped anatase TiO_2_ nanorods). Judging from the orange color of the samples (inset of Figure 4), a black graphitic carbon shell on the surface of TiO_2_ nanorods is unlikely to be formed, which is consistent with the high-resolution TEM characterization result (inset of Figure 3b).

### 2.5. XPS Analysis

**Figure 5:**
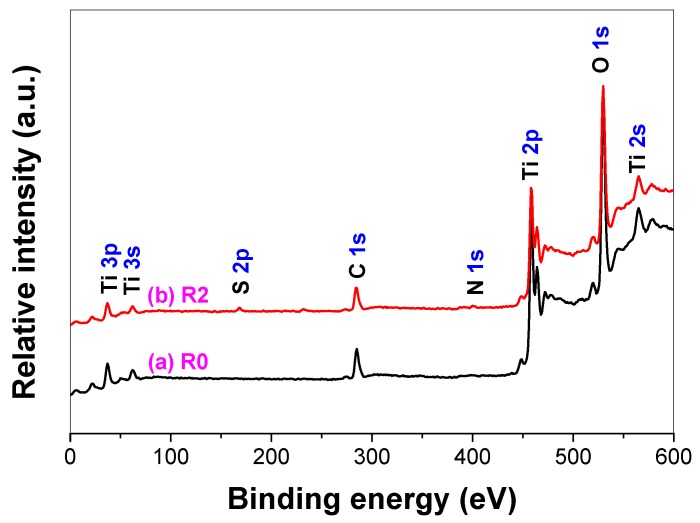
XPS survey spectra of pristine R0 (**a**) and thiourea-modified TiO_2_ nanorods of R2 (**b**).

Figure 5 compares the XPS survey spectra of pristine (R0) and thiourea-modified TiO_2_ nanorods (R2). As demonstrated by XPS, both samples contain Ti, O and C elements. However, small amounts of N and S elements were also detected for thiourea-modified TiO_2_ nanorods.

**Figure 6:**
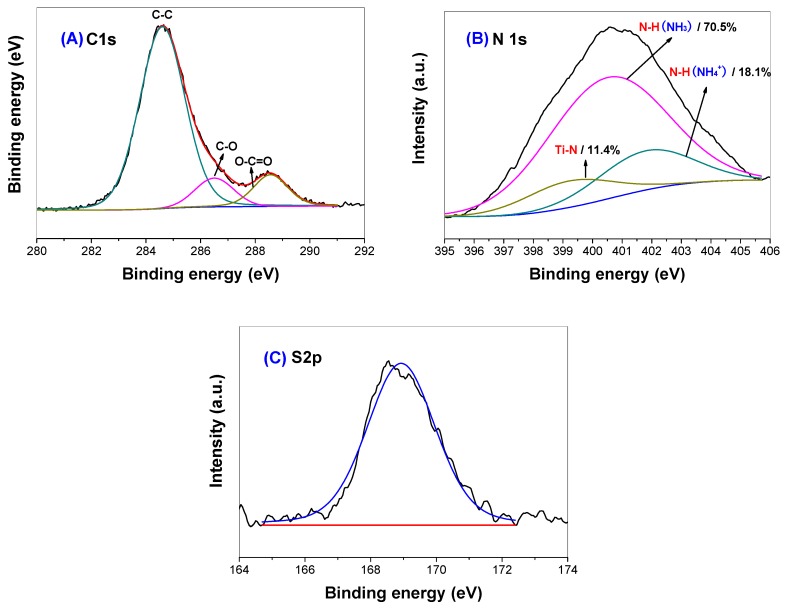
High-resolution XPS spectra of the C 1s (**A**); N 1s (**B**) and S 2p (**C**) regions of R2 sample.

Figure 6 shows the high-resolution XPS spectra of C 1s, N 1s and S 2p regions for thiourea-modified TiO_2_ nanorods, respectively. From Figure 6A, it can be seen that the spectrum of C 1s can be deconvoluted into three peaks, one strong peak and two week peaks at binding energies of 284.6, 286.6 and 288.6 eV, implying three different chemical environments of carbon in the R2 sample. The peak around 284.6 eV is usually assigned to carbon adsorbed on the surface of the photocatalyst as a contaminant, namely C-C bonds, which cannot be eliminated. The peak at 286.6 eV can be ascribed to the existence of C-O bonds, as carbonate species. The peak observed at binding energy of 288.6 eV is attributed to O-C=O bonds [11]. No peak was observed at a binding energy of about 282 eV, corresponding to the incorporation of C atoms in form of Ti-C bond, reflecting that C element was not doped into the lattice of TiO_2_ nanorods [30]. The pertaining peaks in the XPS spectrum may be due to the presence of adventitious carbon species.

Figure 6B shows the corresponding high-resolution XPS spectrum of the N 1s region taken from R2 sample. The curve of the N 1s region can be deconvoluted into three peaks. The small peak (399.2 eV) is attributed to the Ti-N (at. 11.4%). The other two peaks at about 400.5 and 401.8 eV are assigned to NH_3_ (at. 70.8%) and NH_4_^+^ (at. 18.1%) adsorbed on the surface of TiO_2_, respectively [30].

Figure 6C shows the high-resolution XPS spectrum of the S 2p region for R2 sample. It can be seen that the peak of S 2p centers at binding energy of 168.9 eV, which can be attributed to the S (+VI), which is assigned to the SO_4_^2−^ ions adsorbed on the surface of TiO_2_ sample. No obvious signal of the doped S element in the form of Ti-S at binding energy of about 165 eV can be found from the high-resolution XPS spectrum [30]. The failure doping of S element into the lattice of TiO_2_ nanorods may be due to the fact that the diameter of S^2−^ (1.7 Å) ion is larger than that of O^2−^ (1.22 Å) [33].

According to the above XPS results, it can be concluded that only N element was *in situ* doped into the lattice of R2 TiO_2_ nanorods during calcination. The composition of the detected elements by XPS for R0 (pristine TiO_2_ nanorods), R2 and R8 samples are listed in Table 2. It shows that the content of the N and S elements increases with increasing *R* value. However, the content of carbon does increase with increase in *R*, further confirming that the interference of adventitious hydrocarbon is from the XPS instrument itself.

**Table 2:** XPS characterization results of the photocatalysts.

Sample	Molar Ratio			
	C/Ti	N/Ti	S/Ti	O/Ti
R0	2.85	-	-	2.74
R2	2.02	0.079	0.106	3.28
R8	3.69	0.103	0.123	3.81

When compared with our previous reported C, N and S co-doped TiO_2_-HMSs which were prepared by calcination of TiO_2_-HMSs in the presence of cysteine at 300 °C [30], the contents of C, N and S in 600 °C-calcined TiO_2_ nanorods are much lower. This reflects that it is much harder to dope nonmetal ions into the lattice of TiO_2_ at high temperature because C and S elements can be easily oxidized into CO_2_ and SO_2_ in air atmosphere. In addition, the electronegativity of N (3.04) is larger than that of C (2.55) and S (2.58), which also results in an easier N-doping due to the stronger interaction between Ti and N.

It has been reported that anatase titania nanobelts were prepared via hydrothermal processing and subsequent heat treatment in NH_3_ [34]. However, the present method has the merit of being simple and easy to scale up.

### 2.6. Nitrogen Adsorption

**Figure 7:**
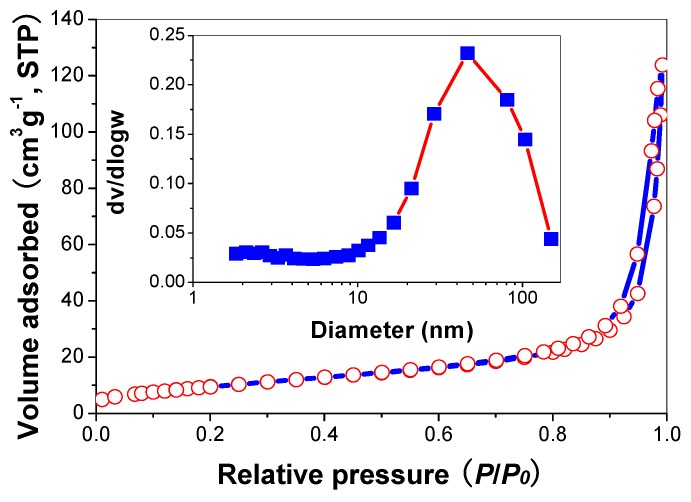
Nitrogen adsorption-desorption isotherm and the corresponding pore size distributions (inset) of R2 sample.

Figure 7 shows the nitrogen sorption isotherms and the corresponding pore size distribution curves of R2 sample. It can be seen that the isotherm is of types IV (BDDT classification) [35]. At high relative pressure range from 0.8 to 1.0, the isotherm exhibits a hysteresis loop of type H2 associated with the ink bottle pores, indicating that the powders contain mesopores (2–50 nm). The corresponding pore size distribution curve of R2 exhibits a wide pore size distribution with the average pore diameters about 12.7 nm.

Table 2 shows the effects of *R* value on the surface properties of the TiO_2_ nanorods. It can be seen that all these samples show similar BET surface areas and pore diameters, reflecting that modification of TiO_2_ nanorods by thiourea has not changed their physical structures.

### 2.7. Photocatalytic Activity

**Figure 8:**
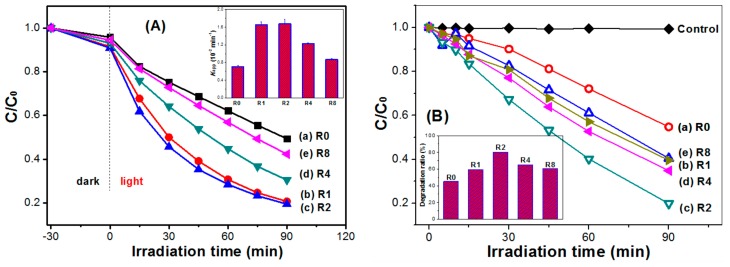
Photocatalytic degradation profiles of X3B (**A**) and RhB (**B**) under visible-light irradiation in the presence of different TiO_2_ nanorods photocatalyst (inset: comparison of the corresponding photocatalytic activity).

**Figure 9:**
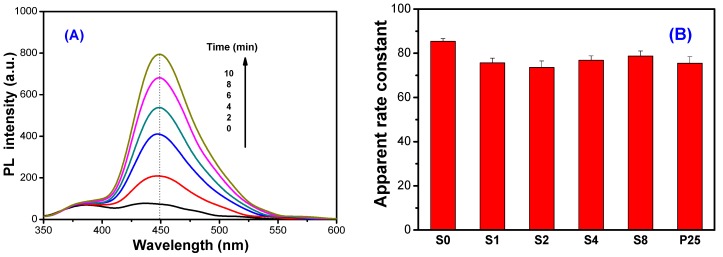
PL spectral changes (**A**) observed during irradiation of the R2 sample in 5.0 × 10^−3^ mol·L^−1^ solution of coumarin (excitation at 332 nm) under UV irradiation, and the comparison of formation rate constant of hydroxyl radicals in the different photocatalyst (**B**).

To study the effect of thiourea modification on the photocatalytic activity of TiO_2_ nanorods, both degradation of X3B (Figure 8A) and RhB (Figure 8B) dye under visible irradiation (Figure 8) and formation of •OH radicals in solution (Figure 9) under UV irradiation were performed. From Figure 8A, it can be seen that pristine R0 shows the smallest adsorption to X3B (only 4.12%), while R2 sample shows the highest adsorption (9.13%). It has been well documented that preliminary adsorption of substrate on the catalyst surface is a prerequisite for highly efficient oxidation [36]. The stronger adsorptive ability of R2 should facilitate the enhancement of its photocatalytic activity. Inset of Figure 8 compares the degradation rate constant of X3B in different photocatalysts. It can be clearly seen that R2 sample shows the highest photocatalytic activity (0.017 min^−1^), which is two times higher than pristine TiO_2_ nanorods (0.0071 min^−1^ for R0). This is consistent with their adsorption capabilities. Similar results were also obtained in photocatalytic degradation of RhB dye. The R2 sample shows the highest degradation ratio of RhB (80.13%), which is almost two times higher than the R0 sample (only 47.27%).

Recently, it has been proved that several non- or weakly luminescent test molecules, such as terephthalic acid [37,38] and coumarin [39,40] produce strongly luminescent compounds with •OH radical, and these molecules can be applied for evaluation of the relative photocatalytic activity of the photocatalyst. Here coumarin is used as a probe to evaluate the photocatalytic activity of TiO_2_ nanorods. Figure 9A shows the typical PL spectral changes observed during illumination of the suspensions of R2 sample. It is observed that the PL intensity of photo-generated 7-hydroxycoumarin at 450 nm (excited at 332 nm) increases with irradiation time, obeying a pseudo-zero order reaction rate equation in kinetics. Figure 9B compares the •OH radical formation rate constant in different photocatalyst. It can be seen that thiourea shows little effect on the UV photocatalytic activity of TiO_2_ nanorods, confirming that the enhanced visible photocatalytic activity of thiourea-modified TiO_2_ nanorods is not based on the sacrifice of their UV photocatalytic activity.

### 2.8. Photoluminescence Spectrum Analysis

**Figure 10:**
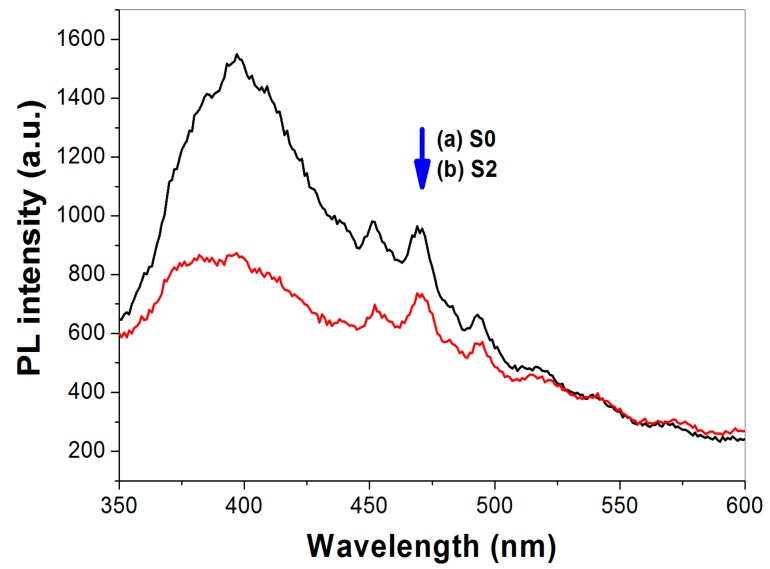
PL spectra of pristine R0 (**a**) and N-doped TiO_2_ nanorods R2 (**b**).

Photoluminescence (PL) analysis is commonly used to analyze the recombination rate of photo-generated electron-hole of semiconductor [41]. Herein, we conduct PL measurement for the pristine (R0) and thiourea-modified TiO_2_ sample (R2), respectively. It can be seen that the emission spectra shapes of R0 and R2 are similar (Figure 10). The strong peak at about 397 nm is attributed to the emission of band gap transition [30]. When compared with pure TiO_2_ nanorods (R0), the intensity of PL signal for R2 is much lower. This is due to the reduction of the radiative recombination process, that is, the lower the recombination, the weaker the PL signals are. Consequently, it is understandable that thiourea-modified TiO_2_ nanorods show superior photocatalytic activity to that of pristine TiO_2_ nanorods (R0).

### 2.9. Photocurrent Response

**Figure 11:**
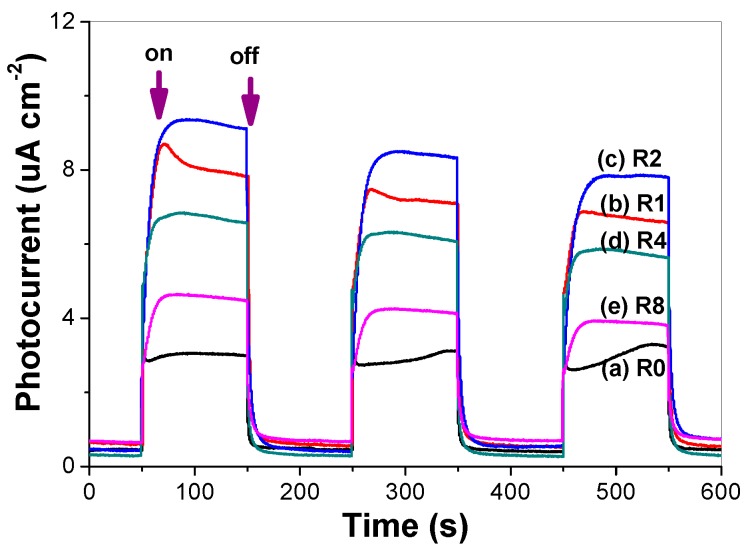
Comparison of the photocurrent response.

The value of photocurrent can indirectly reflect the semiconductor’s ability to generate and transferr photo-generated charge carriers under irradiation [30]. The photocurrent responses of pure and thiourea-modified TiO_2_ nanorods were tested in several on-off cycles (Figure 11). A prompt generation of photocurrents are observed and with good reproducibility when the ITO/TiO_2_ electrodes are illuminated. While the lamp is off, the value of photocurrent for all the ITO/TiO_2_ samples are instantaneously close to zero. It can be clearly seen that the photocurrent value increases first and then decreases with increase in the *R* value, and R2 sample shows the highest photocurrent value, which is consistent with the photocatalytic activity of the samples (Figure 8). The photocatalytic activity of TiO_2_ is highly related to the number of the separated photo-generated charge carriers. Nonmetal doping, in particular nitrogen doping, can be incorporated as a substitutional or insterstitial state in the TiO_2_ lattice, which results in the visible-light activity [6]. From the photocurrent, it can be seen that high concentrated N-doping can cause the formation of photo-generated electron-hole recombination center, which is detrimental to the photocatalytic activity of TiO_2_ nanorods. Therefore, it is understandable that R2 shows superior photocatalytic activity, although R8 possesses the highest visible-light harvesting ability (Figure 4).

## 3. Experimental Section

### 3.1. Preparation

#### 3.1.1. Titanates

In our experiments, the hydrothermal method was employed for the synthesis of titanate nanostructures, which involved a primary reaction between a concentrated NaOH solution and titanium dioxide [16,42]. Briefly, 1.0 g P25 TiO_2_ (Degussa, Shanghai Pharm, China) was dispersed in an 80 mL aqueous solution of NaOH (10 mol·L^−1^), which was then transferred into a 100 mL Teflon-lined autoclave and then oven-heated at 200 °C for 24 h. After cooling to room temperature, the white precipitates were filtrated through a membrane filter (pore size, 0.45 μm) and rinsed with distilled water until the pH value of the filtrate is about 7. The obtained white 1-D sodium titanates cake was re-dispersed in 80 mL of HCl solution (0.1 mol·L^−1^) under magnetic stir. 24 h later, the resulted hydrogen titanates precipitates were washed with deionized water for several times and dried in oven at 80 °C for 6 h.

#### 3.1.2. TiO_2_ Nanorods Precursors

To transformation of the prepared hydrogen titanates to anatase TiO_2_ nanorods, the obtained hydrogen titanates powders were re-dispersed into 80 mL of H_2_SO_4_ solution (0.2 mol·L^−1^), which were then heated at 100 °C for 12 h. Similarly, the obtained white precipitates were collected and dried at 80 °C after wash with deionized water.

#### 3.1.3. N-doped TiO_2_ Nanorods

Certain amount of thiourea (0–3.2 g) was mixed with 0.4 g of the as-prepared TiO_2_ nanorods precursors. The mixture was ground and calcined at 600 °C for 2 h in a furnace. According to this method, the mass ratio of thiourea to TiO_2_ nanorods precursors (*R*) varies from 0 to 8.0. For simplicity, the prepared sample was named as Rx, where x represents *R* value, the mass ratio of thiourea to TiO_2_ nanorods precursors. R0 refers to the calcined TiO_2_ nanorods in the absence of thiourea (Table 1).

### 3.2. Characterization

The X-ray diffraction (XRD) patterns of the samples were obtained on a D8 advance X-ray diffractometer (Germany Bruker, Madison, WI, USA) using Cu Kα radiation at a scan rate (2θ) of 0.05 s^−1^. The voltage and applied current were 40 kV and 80 mA, respectively. The morphology of the photocatalyst was observed on a transmission electron microscopy (TEM) (Tecnai G20, Hillsboro, OR, USA) using an acceleration voltage of 200 kV and a field emission scanning electron microscope (SEM) (Hitach, Tokyo, Japan) with an acceleration voltage of 20 kV, respectively. Nitrogen adsorption-desorption isotherms were obtained on an ASAP 2020 (Micromeritic Instruments, Atlanta, GA, USA) nitrogen adsorption apparatus. All the samples were degassed at 200 °C prior to Brunauer-Emmett-Teller (BET) measurements. The BET specific surface area (*S*_BET_) was determined by a multipoint BET method using the adsorption data in the relative pressure *P*/*P*_0_ range of 0.05–0.30. The desorption isotherm was used to determine the pore size distribution by using the Barret-Joyner-Halenda (BJH) method. The nitrogen adsorption volume at *P*/*P*_0_ = 0.994 was used to determine the pore volume and average pore size. UV-vis diffuse reflectance spectroscopy (DRS) was carried out on a Hitachi U-3010 UV-vis spectrophotometer (Hitachi, Tokyo, Japan). BaSO_4_ was the reference sample. X-ray photoelectron spectroscopy (XPS) measurement was done using Multilab 2000 XPS system (ThermoVG Scientific, East Grinstead, West Sussex, UK) with a monochromatic Mg Kα source and a charge neutralizer, all the binding energies were referenced to the C 1s peak at 284.6 eV of the surface adventitious carbon. Photoluminescence (PL) spectra were measured at room temperature on a Fluorescence Spectrophotometer (F-7000, Hitachi, Tokyo, Japan). The excitation wavelength was 315 nm, the scanning speed was 1200 nm·min^−1^, and the PMT voltage was 700 V. The width of excitation slit and emission slit were both 5.0 nm.

### 3.3. Photocatalytic Activity

#### 3.3.1. Visible Photoctalytic Activity

A 350-W Xe lamp (Lanshen electronics, Shanghai, China) used as the light source was positioned above a cylindrical Pyrex vessel surrounded by a jacket with circulating water. A cutoff filter was used to remove wavelengths less than 420 nm completely and to ensure irradiation only by visible light. Brilliant Red X-3B (X3B) [43,44,45], an anionic azo dye, is used as a probe molecule. During the photocatalytic reaction, the reactor was mechanically stirred at a constant rate. The concentration of TiO_2_ was 1.0 g·L^−1^, and the initial concentration of X3B was 1.0 × 10^−4^ mol·L^−1^. Before irradiation, the suspensions were sonicated first for 5 min, and then were shaken overnight in the dark to established the adsorption-desorption equilibrium. At given intervals of irradiation, small aliquots were withdrawn by a syringe, and filtered through a membrane (pore size 0.45 μm). The concentration of X3B remaining in the filtrate was then analyzed by an Agilent 8451 spectrometer (Aglent Technologies, Palo Alto, CA, USA) at 510 nm.

Photocatalytic activity degradation of Rhodamine B (RhB) dye was also performed similar to the degradation of X3B. The initial concentration of RhB for degradation is 1.0 × 10^−5^ mol·L^−1^, and the wavelength for analysis of RhB is 554 nm.

#### 3.3.2. UV Photocatalytic Activity

The UV photocatalytic activity of the photocatalyst was evaluated by a photoluminescence (PL) technique using coumarin as a probe molecule, which readily reacted with •OH radicals to produce highly fluorescent product, 7-hydroxycoumarin, under UV irradiation [39]. The suspensions of TiO_2_(1.0 g·L^−1^) containing coumarin (0.5 mmol·L^−1^) is mixed under magnetic stirring, and then was shaken overnight. At given intervals of irradiation, small aliquots were withdrawn by a syringe, and filtered through a membrane (pore size 0.45 μm). Solution after filtration was analyzed on a Hitachi F-7000 fluorescence spectrophotometer by the excitation with the wavelength of 332 nm.

### 3.4. Photocurrent

Photocurrent measurements were carried out on an Electrochemical Station (CHI660, Chenhua Instrument, Shanghai, China). The Xe lamp with cutoff filter was used for excitation of the ITO/TiO_2_ electrode. These measurements were carried out with a standard three-electrode assembly. An ITO/TiO_2_ electrode, Pt plate, and Ag/AgCl electrode were used as the working, counter, and reference electrodes, respectively. The ITO/TiO_2_ electrode was prepared using a doctor-blade method. Na_2_SO_4_ (0.4 mol·L^−1^) is used as the electrolyte and is saturated with air.

## 4. Conclusions

Enhanced visible photocatalytic activity of TiO_2_ nanorods were successfully studied through thiourea modification of TiO_2_ by calcination the mixture of TiO_2_ nanorods and thiourea at 600 °C. Only N was doped into the lattice of TiO_2_ nanorods due to the greater electronegativity of N (3.04) than that of C (2.55) and S (2.58). The enhanced photocatalytic activity of the R2 sample is due to the synergistic effects of improved crystallization and increased light-harvesting ability, which results in the reduced recombination of photo-generated electron-hole pairs. The enhanced visible photocatalytic activity of thiourea-modified TiO_2_ nanorods is not based on the sacrifice of their UV photocatalytic activity.
